# Cardiotoxicity of Antitumor Agents: Therapeutic Challenges in Heart Failure with Reduced and Preserved Ejection Fraction

**DOI:** 10.3390/ijms27072973

**Published:** 2026-03-25

**Authors:** Marco Tana, Rachele Piccinini, Giada Pinterpe, Ettore Porreca, Rossana Berardi, Claudio Tana

**Affiliations:** 1 Internal Medicine and Cardiovascular Ultrasound Unit, Medical Department, Saint Annunziata Hospital, 66100 Chieti, Italy; 2 School of Internal Medicine, Faculty of Medicine, G. D’Annunzio University, 66100 Chieti, Italy; 3 School of Oncology Diseases, Polytechnic University of Marche, 60131 Ancona, Italy; 4 Internal Medicine Unit, ASL Taranto, 74121 Taranto, Italy

**Keywords:** cardio-oncology, heart failure, cardiotoxicity, anthracyclines, immune checkpoint inhibitors, strain imaging, cardioprotection

## Abstract

The remarkable evolution of oncological therapies has dramatically improved cancer survival rates but has simultaneously introduced a significant burden of cardiovascular complications. Cardio-oncology has emerged as a critical multidisciplinary field focused on mitigating the “collateral damage” of life-saving anticancer treatments, ranging from traditional chemotherapeutics to novel immunotherapies. This review provides a comprehensive analysis of the pathophysiological mechanisms, clinical phenotypes, and evolving management strategies for cancer therapy-related cardiac dysfunction (CTRCD). An extensive synthesis of the current literature was conducted, focusing on the molecular pathways of cardiotoxicity, including Topoisomerase IIβ inhibition by anthracyclines, HER2 signaling disruption by targeted agents, and immune-mediated myocarditis triggered by checkpoint inhibitors (ICIs). Cardiotoxicity is increasingly recognized as a spectrum of phenotypes. Heart failure with reduced ejection fraction (HFrEF) remains a primary concern with cytotoxic agents, while heart failure with preserved ejection fraction (HFpEF) is emerging as a critical complication of radiation therapy and tyrosine kinase inhibitors (TKIs). The integration of advanced diagnostic tools—specifically Global Longitudinal Strain (GLS) and Cardiac Magnetic Resonance (CMR) mapping—has shifted the clinical focus toward subclinical detection. Furthermore, pivotal clinical trials such as PRADA and SUCCOUR have validated early pharmacological prophylaxis and strain-guided interventions. Emerging challenges, including the management of CAR-T cell-induced cytokine release syndrome and the specific cardiovascular needs of pediatric and geriatric populations, are also explored. The future of cardio-oncology lies in precision medicine, leveraging genomic profiling and artificial intelligence to identify high-risk individuals. A proactive, multidisciplinary approach is essential to ensure that the success of modern oncology is not compromised by irreversible cardiovascular morbidity.

## 1. Introduction

The formal recognition of cardio-oncology as a distinct clinical entity is relatively recent, yet its roots date back to the late 1960s and early 1970s. The first major milestone occurred with the introduction of anthracyclines, specifically daunorubicin and doxorubicin, into clinical practice. While these agents showed unprecedented efficacy against leukemias and solid tumors, clinicians quickly noticed a paradoxical increase in severe, often fatal, heart failure among survivors. Initial clinical observations by Lefrak et al. in 1973 [1] were among the first to systematically describe the dose-dependent nature of anthracycline-induced cardiomyopathy, establishing the concept of “cumulative cardiotoxicity”.

Throughout the 1980s, the discovery of the cardioprotective potential of dexrazoxane and the development of endomyocardial biopsy techniques allowed for a deeper histological understanding of myofibrillar dropout and vacuolization. However, it was not until the late 1990s, with the advent of trastuzumab (the first HER2-targeted therapy), that the field realized cardiotoxicity was not limited to direct cytotoxic cell death but could also arise from the interruption of essential cardiac signaling pathways. This led Ewer et al. [2] to propose the classic distinction between Type I (irreversible) and Type II (reversible) cardiac injury, a classification that remains a foundational pillar of cardio-oncological education today. These early discoveries shifted the oncological focus from simple tumor eradication to a more balanced approach that considers the “collateral damage” to the cardiovascular system.

The growing success of cancer therapies has significantly increased patient survival, bringing attention to long-term cardiovascular complications caused by these treatments [3,4]. Cardio-oncology has thus emerged to address the management of heart damage related to anticancer drugs, focusing on prevention, early detection, and treatment of cardiotoxicity [5,6]. This paradigm shift is driven by the realization that cardiovascular disease is now a leading cause of morbidity and mortality among cancer survivors, sometimes exceeding the risk of cancer recurrence itself.

Anticancer agents, including chemotherapy, targeted therapies, and immunotherapies, can induce cardiac dysfunction ranging from heart failure with reduced ejection fraction (HFrEF) to heart failure with preserved ejection fraction (HFpEF) [7,8]. HFrEF typically results from direct myocardial injury by drugs like anthracyclines, while HFpEF involves complex mechanisms such as systemic inflammation and microvascular dysfunction often exacerbated by comorbidities common in cancer patients [2,9].

Given the complexity of cardiotoxic effects and their impact on cancer care, a multidisciplinary approach involving oncologists and cardiologists is essential [10]. Early risk stratification and cardioprotective strategies are key to improving outcomes [11,12,13]. Ongoing research into biomarkers and therapies aims to reduce cardiac risks without compromising cancer treatment efficacy [14]. This review explores the pathophysiology of cardiotoxicity induced by cancer treatments, the clinical features of associated heart failure, and current therapeutic options, aiming to support better integrated patient care in this evolving field [15,16] [Table 1].

### Research Objective and Literature Search Strategy

The primary objective of this review is to provide a comprehensive and updated analysis of the molecular mechanisms, clinical manifestations, and management strategies of cancer therapy-related cardiac dysfunction (CTRCD). Unlike existing narrative reviews, this work specifically addresses the “multimodal cardiotoxicity” of 2026, integrating classic cytotoxic injury with novel immune-mediated and targeted therapy-related challenges.

To ensure transparency and reproducibility, a structured literature search was conducted in accordance with the PRISMA (Preferred Reporting Items for Systematic Reviews and Meta-Analyses) guidelines. We searched PubMed/MEDLINE, Scopus, and Web of Science for articles published between January 2015 and January 2026. Search terms included: “cardio-oncology”, “ICI-myocarditis”, “GLS-guided therapy”, “BTK-inhibitors cardiotoxicity”, “RARG pediatric cardiotoxicity”, and “AI in heart failure oncology”.

Inclusion Criteria:

- Randomized controlled trials (RCTs) and meta-analyses.
- International guidelines (ESC 2022, ASCO 2017) [4,30].
- Observational studies with a minimum follow-up of 12 months.
- Peer-reviewed articles in English.

Exclusion Criteria:

- Case reports and small case series (n < 10).
- Non-peer-reviewed conference abstracts.
- Studies focused exclusively on pre-clinical animal models without clinical translation.

**Figure 1:**
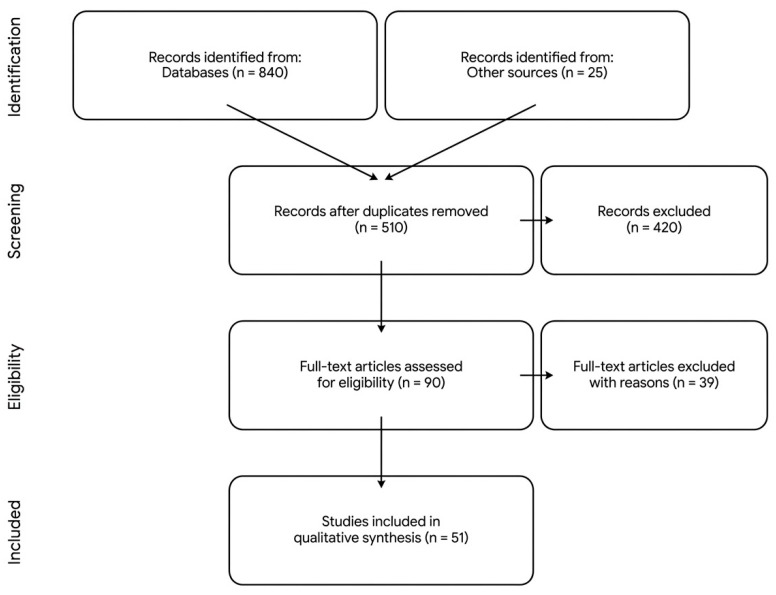
PRISMA 2020 flow diagram of the systematic literature search.

The study selection process, including identification, screening, and inclusion, is summarized in the PRISMA flow diagram (Figure 1).

The flow diagram illustrates the systematic process of identification, screening, eligibility, and inclusion of studies for this review. From an initial identification of 865 records across three electronic databases (PubMed/MEDLINE, Scopus, and Web of Science) and secondary sources, 51 studies met the pre-defined inclusion criteria. The selection focused on clinical trials, international guidelines (ESC/ASCO), and high-impact systematic reviews published between 2015 and 2026 to ensure an updated evidence base for the cardiovascular management of cancer patients.

## 2. Mechanisms of Chemotherapy-Induced Cardiotoxicity

The pathophysiology of drug-induced cardiotoxicity involves a complex network of molecular pathways (Table 1) [21,30,31]. The increasing number of cancer survivors, now exceeding 18 million in the US alone [3], underscores the urgency of understanding these mechanisms to mitigate long-term cardiovascular morbidity.

### 2.1. Anthracyclines: Topoisomerase II-Beta (Top2b) and Redox Dysregulation

While early models focused on the generation of reactive oxygen species (ROS) via the quinone moiety of doxorubicin, the modern paradigm centers on the interaction with Topoisomerase II-beta (Top2b). In malignant cells, anthracyclines target Topoisomerase II-alpha (Top2a) to inhibit proliferation; however, cardiomyocytes constitutively express the Top2b isoform. Anthracyclines stabilize a transient covalent intermediate, known as the “cleavable complex,” involving Top2b and double-stranded DNA. This interaction prevents DNA religation and leads to persistent double-strand breaks (DSBs) [4,5,6,7]. These downstream effects are significant, as the Top2b-doxorubicin complex triggers the activation of p53 and the subsequent downregulation of genes involved in mitochondrial biogenesis, such as PGC-1α e PGC-1β. This cascade leads to a profound bioenergetic crisis, impaired mitophagy, and ultimately, cardiomyocyte apoptosis [3,4,31].

### 2.2. HER2/ErbB2 Signaling Disruption

Trastuzumab-induced cardiotoxicity is primarily driven by the inhibition of the Neuregulin-1 (NRG1)/ErbB signaling axis, which is a vital survival pathway for the heart. Under physiological conditions, NRG1 binds to ErbB4, promoting its heterodimerization with ErbB2, a pathway essential for sarcomere assembly and cardiomyocyte repair during hemodynamic stress [16]. By sequestering ErbB2, Trastuzumab disrupts this critical survival signal, leading to the downregulation of the PI3K/Akt and MAPK/ERK pathways. Unlike the irreversible damage caused by anthracyclines, this signaling blockade usually results in contractile dysfunction—often described as myocardial hibernation—rather than immediate cell death, frequently allowing for functional recovery upon drug discontinuation [3,16].

### 2.3. VEGF Inhibitors and Tyrosine Kinase Inhibitors (TKIs)

The “off-target” effects of TKIs, such as Sunitinib and Sorafenib, often result in a phenotype of Heart Failure with preserved Ejection Fraction (HFpEF) through a combination of vascular and cellular insults. Endothelial dysfunction plays a central role, as the inhibition of Vascular Endothelial Growth Factor (VEGF) receptors reduces the activity of eNOS (endothelial Nitric Oxide Synthase), thereby decreasing Nitric Oxide (NO) bioavailability. This results in systemic vasoconstriction and hypertension, which significantly increases left ventricular (LV) afterload [14]. Simultaneously, these agents cause bioenergetic impairment; some TKIs directly inhibit AMPK (AMP-activated protein kinase), leading to a reduction in ATP production and the induction of endoplasmic reticulum (ER) stress, which promotes myocardial stiffening and diastolic dysfunction [3,30].

### 2.4. Immune Checkpoint Inhibitors (ICIs) and CAR-T Cells

The mechanism of ICI-induced myocarditis is characterized by acute, high-grade inflammatory infiltration due to the loss of immune tolerance. The inhibition of PD-1, PD-L1, or CTLA-4 pathways leads to the activation of T-lymphocytes that may target myocardial antigens due to molecular mimicry with tumor cells [30]. This T-cell activation triggers a potent cytokine storm, where the infiltration of CD4+ and CD8+ T-cells is accompanied by a massive release of pro-inflammatory cytokines, including IFN-γ, TNF-α, and IL-6. This cascade leads to rapid cardiomyocyte necrosis and is often associated with lethal conduction disturbances or arrhythmias, representing one of the most acute challenges in modern cardio-oncology [30].

## 3. Heart Failure Phenotypes: HFrEF and HFpEF in Oncology Patients

Heart failure in oncology patients typically manifests in two principal phenotypes—heart failure with reduced ejection fraction (HFrEF) and heart failure with preserved ejection fraction (HFpEF)—which often represent distinct biological responses to different classes of anticancer agents and radiotherapy (Table 2).

### 3.1. HFrEF (Heart Failure with Reduced Ejection Fraction)

HFrEF (LVEF < 40%) is often a direct consequence of overt cardiomyocyte injury, involving structural loss and architectural collapse of the myocardium. Anthracyclines and targeted therapies like trastuzumab are strongly associated with this phenotype, though they act through different pathways. Anthracyclines induce mitochondrial damage and massive ROS generation, leading to myofibrillar dropout and a permanent impairment of contractile function. Trastuzumab, while often reversible, disrupts the neuregulin-1/ErbB2 signaling essential for sarcomeric repair. This phenotype is commonly symptomatic, presenting with classic signs of congestion, and is primarily detected through significant echocardiographic reductions in LVEF. Its management necessitates the early initiation of standard HF therapy, including β-blockers, ACE inhibitors, or ARNI, to mitigate progressive remodeling [2,17,34,35].

### 3.2. HFpEF (Heart Failure with Preserved Ejection Fraction)

HFpEF (LVEF ≥ 50%) is increasingly recognized as a major cause of morbidity in cancer survivors, particularly among older individuals and those with a high burden of metabolic comorbidities. It is characterized by diastolic dysfunction, increased ventricular stiffness, and impaired myocardial relaxation, even in the presence of a normal pumping capacity. Unlike the cellular attrition seen in HFrEF, HFpEF is associated with extensive myocardial remodeling, interstitial fibrosis, and systemic inflammation [36]. Cancer therapies, particularly mediastinal radiation and certain TKIs, can promote chronic endothelial dysfunction and microvascular rarefaction. These processes lead to impaired nitric oxide bioavailability and increased collagen deposition, which collectively enhance myocardial stiffness and elevate left ventricular filling pressures [11,32].

## 4. Immunotherapy and CAR-T Cell Therapy: Cardiovascular Implications

A new frontier in cardio-oncology is the management of Chimeric Antigen Receptor (CAR) T-cell therapy, a revolutionary form of adoptive immunotherapy that has transformed the prognosis for refractory hematological malignancies, such as B-cell acute lymphoblastic leukemia and non-Hodgkin lymphomas [37]. This therapy involves a sophisticated genetic engineering process: a patient’s own T-lymphocytes are extracted and modified ex vivo to express a synthetic receptor (the CAR) designed to recognize specific tumor antigens, such as CD19, independently of the major histocompatibility complex (MHC). Once re-infused into the patient, these “living drugs” undergo rapid clonal expansion and execute a targeted cytotoxic strike against malignant cells. However, the unprecedented efficacy of CAR T-cells comes with a unique set of cardiovascular challenges.

The primary concern is Cytokine Release Syndrome (CRS), a systemic inflammatory response triggered by the massive activation of CAR T-cells and bystander immune cells, such as macrophages. This results in an exponential surge of pro-inflammatory mediators, particularly interleukin-6 (IL-6), interferon-gamma (IFN-γ), and tumor necrosis factor-alpha (TNF-α) [10,24]. From a cardiovascular perspective, this immune storm translates into a state of distributive shock characterized by profound peripheral vasodilation and increased capillary permeability, often referred to as capillary leak. More critically, high levels of IL-6 exert a direct, acute suppressive effect on myocardial contractility by modulating calcium handling and mitochondrial function, potentially triggering arrhythmias, localized pericarditis, or a stress-induced cardiomyopathy that mimics Takotsubo syndrome [37].

### Management of CAR-T Related Toxicity

Management of cardiac complications during immunotherapy requires a tiered, rapid-response approach to prevent irreversible damage, as outlined in the 2022 ESC Guidelines on Cardio-Oncology [4]. Grade 1–2 CRS is primarily managed with tocilizumab, a monoclonal antibody that blocks the IL-6 receptor, often resulting in rapid hemodynamic stabilization. However, when cardiac dysfunction progresses to Grade 3 or 4, the clinical scenario becomes life-threatening. For professionals from other fields, Grade 3 is characterized by severe heart failure symptoms (NYHA Class III–IV) or a significant drop in left ventricular ejection fraction (LVEF < 40%), while Grade 4 involves cardiogenic shock requiring urgent vasopressor support or mechanical circulatory assistance. In these high-grade cases, high-dose corticosteroids are mandatory to provide a broader immunosuppressive effect. Methylprednisolone, administered intravenously at 1–2 mg/kg/day, is the preferred agent due to its rapid action, although Dexamethasone (10 mg every 6 h) is frequently utilized if there is concomitant neurological involvement [23,24].

Given the rapid onset of these complications, continuous telemetry and daily monitoring of cardiac biomarkers, such as NT-proBNP and Troponin, during the “peak” cytokine window—usually occurring between days 3 and 10 post-infusion—is mandatory to facilitate early intervention [14,24]. It is crucial to note that while these biomarkers are highly sensitive, they are not pathognomonic for CAR-T related toxicity, as elevations can be seen in sepsis or pulmonary embolism. Therefore, a rigorous differential diagnosis is necessary, incorporating more specific markers such as a reduction in Global Longitudinal Strain (GLS) greater than 15% [18] or the presence of myocardial edema and inflammation on Cardiac Magnetic Resonance (CMR) [36,38]. While basic monitoring can be performed at most medical levels, the management of Grade 3–4 toxicities and the use of advanced imaging like GLS and CMR require the specialized infrastructure of tertiary or quaternary cardio-oncology units [4,5].

## 5. Cardiovascular Complications in Pediatric and Geriatric Populations

### 5.1. Pediatric Cardio-Oncology: Growth and Remodeling

Survivors of childhood cancers represent an exceptionally unique and vulnerable population due to the protracted and complex interaction between early cytotoxic injury and active physiological development (Table 3). Anthracycline exposure during these critical stages of childhood development does not merely cause acute damage but leads to the onset of a specific “thin-walled” cardiomyopathy, a condition fundamentally distinct from the cardiotoxic phenotypes observed in adults. This pathology is characterized by a significant failure in myocardial mass gain; as the child undergoes rapid somatic growth, the heart remains unable to increase its ventricular mass proportionally to the increasing body surface area (BSA). This critical mismatch between cardiac size and physical stature results in a state of chronically elevated afterload and high systolic wall stress, which progressively impairs cardiac compliance and diastolic filling [26,30].

As detailed in the Childhood Cancer Survivor Study, these patients are at significantly higher risk for late-onset Heart Failure with reduced Ejection Fraction (HFrEF), a complication that often manifests insidiously decades after the successful completion of oncological treatment [26]. From a pathophysiological standpoint, this structural deficit creates a permanent mass-to-volume imbalance, preventing the heart from meeting the increasing metabolic and hemodynamic demands of an adult body. Consequently, these survivors face an accelerated decline in cardiac reserve and a heightened susceptibility to secondary cardiovascular insults later in life [30,35]. Furthermore, recent advancements in cardio-genomics have elucidated that this susceptibility is often exacerbated by specific genetic predispositions, such as coding variants in the RARG gene, which sensitize cardiomyocytes to anthracycline-induced damage [19,40].

Given the progressive and often subclinical nature of this structural remodeling, clinical surveillance must be proactive rather than reactive. Lifelong monitoring protocols utilizing serial echocardiography or, more ideally, Cardiac Magnetic Resonance (CMR) are essential to track the ventricular mass-to-BSA ratio accurately. This allows for the timely initiation of cardioprotective therapies before the transition to overt heart failure, ensuring that the long-term cardiovascular health of pediatric cancer survivors is preserved throughout their adult lives [19,30,38].

### 5.2. Geriatric Cardio-Oncology: The Complexity of Comorbidities

In elderly patients, the cardiovascular system is frequently already compromised by age-related structural changes, such as increased arterial stiffening, left ventricular hypertrophy, and reduced beta-adrenergic sensitivity (Table 3) [11,16]. These baseline physiological alterations create a fragile substrate where modern cancer therapies often act as a catalyst that unmasks latent Heart Failure with preserved Ejection Fraction (HFpEF) or exacerbates pre-existing diastolic dysfunction [41]. Unlike younger cohorts, the geriatric demographic presents a significant prevalence of pre-existing cardiovascular diseases, such as coronary artery disease and calcific valvular heart disease, which significantly lowers the threshold for therapy-related cardiac injury [39].

The clinical management of geriatric patients requires a delicate balance; clinicians must address the high risk of polypharmacy and potential drug–drug interactions, particularly between novel targeted agents and essential medications like oral anticoagulants, anti-hypertensives, and statins, while ensuring that the intensity of cancer treatment remains effective [27,41]. A critical aspect of this pharmacological complexity is the shared metabolism of many small-molecule Tyrosine Kinase Inhibitors (TKIs) and supportive drugs via the cytochrome P450 (CYP3A4) pathway, which can lead to unpredictable plasma concentrations and heightened toxicity [27].

Furthermore, according to the 2022 ESC Guidelines on Cardio-Oncology, the use of standardized risk stratification tools, such as the HFA-ICOS score, is paramount in this population to identify high-risk individuals before the initiation of potentially cardiotoxic regimens [4,30]. The goal is to implement a “proactive” management strategy where aggressive control of cardiovascular risk factors—including hypertension and dyslipidemia—is integrated into the oncological care plan, thereby reducing the incidence of acute cardiovascular events and preventing the premature discontinuation of life-saving cancer therapies [4,9,41].

## 6. Electrophysiology: Arrhythmias and Ibrutinib

Ibrutinib, a first-in-class Bruton tyrosine kinase (BTK) inhibitor, has significantly improved outcomes in B-cell malignancies but is associated with a notable incidence of atrial fibrillation (AF) and a complex profile of hemorrhagic complications. The cardiotoxicity of Ibrutinib is primarily driven by the “off-target” inhibition of C-terminal Src kinase (CSK) in the myocardium, a protein essential for maintaining cardiac electrophysiological stability. This inhibition alters calcium handling within cardiomyocytes, creating a pro-arrhythmogenic substrate that significantly increases the risk of AF, with an incidence reaching up to 15% in treated cohorts [27,42].

The clinical management of these patients is further complicated by a unique “thrombo-hemorrhagic paradox” (Table 4). While Ibrutinib promotes the embolic risk associated with AF, it simultaneously inhibits platelet aggregation through the blockade of BTK and Tec kinases, which are critical signaling components for collagen-induced platelet activation [27]. This dual effect creates a significant challenge for clinicians, as the patient is at high risk for both thromboembolic events and mucocutaneous bleeding, particularly when anticoagulation is initiated. According to current management algorithms, the choice of anticoagulation must be carefully balanced, often preferring non-vitamin K antagonist oral anticoagulants (NOACs) while vigilantly monitoring for drug–drug interactions [27,28].

As summarized in Table 4, Ibrutinib metabolism occurs predominantly via the cytochrome P450 (CYP3A4) pathway. This shared metabolic route with many common cardiovascular medications, such as diltiazem, verapamil, and certain azole antifungals, can lead to dangerous fluctuations in drug plasma levels and heightened toxicity [27]. Furthermore, the evolution of BTK inhibitors has led to the development of second-generation agents like Acalabrutinib and Zanubrutinib, which exhibit higher selectivity for BTK and reduced off-target inhibition of CSK and Tec. These newer molecules offer a more favorable safety profile, with a significantly lower incidence of atrial fibrillation and bleeding compared to Ibrutinib [27]. This distinction underscores the importance of a personalized approach in cardio-oncology, where the selection of the specific inhibitor must be guided by the patient’s baseline cardiovascular risk and the potential for long-term complications [4,27].

## 7. Vascular Toxicities and Radiation-Induced Valvular Disease

Radiation therapy remains a cornerstone in the treatment of various malignancies, including breast cancer, Hodgkin lymphoma, and lung cancer. However, the incidental exposure of the heart to ionizing radiation can lead to a spectrum of cardiovascular complications, collectively known as Radiation-Induced Heart Disease (RIHD) (Table 5). The pathophysiology of RIHD is fundamentally distinct from chemotherapy-induced injury, as it primarily involves microvascular damage, accelerated atherosclerosis of the coronary arteries, and progressive myocardial fibrosis [15,23].

As established in the landmark study by Darby et al. [15], the risk of major coronary events increases linearly with the mean dose of radiation to the heart, with no clear threshold below which the risk is eliminated. Specifically, the study demonstrated that the rate of major coronary events increases by 7.4% per Gray (Gy) of mean heart dose, and this risk persists for decades after exposure [15]. Furthermore, radiation-induced injury can affect the heart valves, typically leading to calcification and stenosis (most commonly the aortic and mitral valves), as well as the conduction system, resulting in various degrees of heart block or arrhythmias [4,15,43].

The modern approach to RIHD emphasizes advanced radiotherapy techniques, such as Deep Inspiration Breath Hold (DIBH) and Intensity-Modulated Radiation Therapy (IMRT), which aim to minimize the mean heart dose (MHD). According to the 2022 ESC Guidelines, long-term clinical surveillance is mandatory for survivors who received significant cardiac radiation, particularly those with pre-existing cardiovascular risk factors or those treated with concomitant cardiotoxic chemotherapy [4]. This monitoring strategy, summarized in Table 5, integrates periodic clinical evaluation with advanced imaging to detect late-onset valvular disease or coronary artery disease before they become symptomatic [4,23,29,30].

## 8. Clinical Trial Evidence and Prophylaxis

The current evidence-based framework for cardio-oncology is guided by several pivotal randomized controlled trials that have redefined the timing and nature of intervention (Table 6). The PRADA trial provided critical insights into pharmacological prophylaxis, demonstrating that the angiotensin receptor blocker candesartan could effectively attenuate the decline in left ventricular ejection fraction (LVEF) during anthracycline-based regimens, whereas the beta-blocker metoprolol showed a more significant effect on reducing biomarkers of myocardial injury rather than preserving volumetric function [44,45] especially in patients with preexisting cardiovascular disease [46].

Similarly, the SUCCOUR trial represented a milestone in diagnostic-led therapy, validating that a management strategy guided by Global Longitudinal Strain (GLS)—specifically starting cardioprotection when a relative reduction in strain (>15%) is detected—prevents a further decline in LVEF and the development of overt heart failure compared to traditional monitoring [18,47]. Furthermore, the CECCY trial supported the early use of carvedilol in patients receiving doxorubicin, highlighting its role in reducing subclinical myocardial injury and improving diastolic parameters, even when the primary endpoint of LVEF preservation was not fully met [48,49]. Collectively, these studies underline the clinical imperative of a “preventative” approach, shifting the focus toward starting cardioprotective therapy at the first sign of subclinical damage, well before the ejection fraction drops below the threshold of clinical heart failure [4,12,13].

### Guidelines for Monitoring and Management of Suspected Cardiotoxicity

Current international guidelines, primarily the 2022 ESC Guidelines on Cardio-Oncology and the ASCO Clinical Practice Guidelines, emphasize a structured approach to monitoring oncology patients [4,30]. Surveillance should be guided by the baseline cardiovascular risk, which is assessed using validated tools like the HFA-ICOS score [4,39]. In the presence of a suspected cardiotoxic event—defined by an asymptomatic decline in LVEF by >10 percentage points to a value < 50%, or a significant rise in cardiac biomarkers—prompt diagnostic intensification is mandatory [4,38].

If cardiotoxicity is confirmed, the initiation of heart failure therapies, including ACE-inhibitors and beta-blockers, is recommended to facilitate myocardial recovery and allow for the safe continuation of the oncological regimen [12,13]. For specific agents like Immune Checkpoint Inhibitors, a suspicion of myocarditis necessitates the immediate cessation of therapy and the introduction of high-dose corticosteroids, followed by advanced imaging such as Cardiac Magnetic Resonance (CMR) to characterize myocardial edema and fibrosis [24,37]. This multi-modality approach ensures that cardiovascular health is maintained without compromising the efficacy of cancer treatment [5,6].

## 9. Current Guidelines for Cardio-Monitoring and Management of Suspected Cardiotoxicity

The clinical surveillance of oncology patients has been standardized by recent international consensus to transition from a reactive model to a proactive, risk-based strategy (Table 7). Central to this framework are the 2022 ESC Guidelines on Cardio-Oncology and the ASCO Clinical Practice Guidelines, which mandate that the intensity of monitoring be dictated by the patient’s baseline cardiovascular risk, determined through validated tools such as the HFA-ICOS assessment [4,29,30]. This structured approach ensures that high-risk individuals receive frequent biochemical and imaging evaluations to detect subclinical injury before it progresses to overt heart failure.

In clinical practice, a suspicion of cardiotoxicity is often triggered by an asymptomatic decline in LVEF (typically a drop of >10 percentage points to a value below 50%) or a significant elevation in cardiac biomarkers such as high-sensitivity Troponin and NT-proBNP [4,14,23]. When such a suspicion arises, a specialized diagnostic workup is mandatory. This includes the use of Global Longitudinal Strain (GLS) to identify subtle myocardial deformation and Cardiac Magnetic Resonance (CMR) to characterize tissue-level changes, such as inflammatory edema or replacement fibrosis [24,36,38]. If cardiotoxicity is confirmed, the immediate initiation of evidence-based heart failure therapies—primarily ACE-inhibitors and beta-blockers—is recommended to facilitate myocardial recovery and ensure that life-saving oncological treatments can be safely continued [12,13,48].

For specialized therapies like Immune Checkpoint Inhibitors (ICIs), the clinical suspicion of myocarditis represents a medical emergency that requires the immediate cessation of the drug and the administration of high-dose corticosteroids, followed by continuous telemetry and daily biomarker monitoring [23,24,37]. This integrated management strategy, which balances the need for aggressive cancer therapy with the preservation of hemodynamic stability, is now recognized as the gold standard in cardio-oncological care, necessitating a multidisciplinary team to manage the most complex cases [4,5,6].

## 10. Future Perspectives: AI and Precision Medicine

The integration of Artificial Intelligence (AI) and machine learning represents the definitive frontier in the personalization of cardio-oncological care, addressing the clinical need to synthesize vast datasets from multi-modality monitoring into actionable insights (Table 8). Contemporary literature highlights that deep learning algorithms can significantly refine the precision of echocardiographic assessments, particularly in automated chamber segmentation and the prediction of left ventricular ejection fraction (LVEF) decline. These models often outperform manual human assessments by reducing inter-observer variability and increasing the speed of analysis in high-volume clinical cohorts [25,32,50,51].

A pivotal application of AI in this field is the automated analysis of Global Longitudinal Strain (GLS) through advanced speckle-tracking software. By providing real-time, highly reproducible measurements of myocardial deformation, AI-driven tools facilitate the early identification of subclinical cardiotoxicity, enabling the timely initiation of cardioprotective therapies as demonstrated in the SUCCOUR trial [18,32]. Beyond imaging, machine learning models are being developed to create integrated polygenic risk scores. These algorithms can process complex “omics” data to identify specific genetic susceptibilities, such as the RARG variant that predisposes pediatric survivors to anthracycline-induced damage, effectively transitioning the field toward a model of precision medicine [19,40].

Furthermore, the emerging field of radiomics—the high-throughput extraction of quantitative features from standard medical images—allows AI to detect subtle myocardial and vascular alterations that are invisible to the naked eye. In patients undergoing radiation therapy, AI-assisted analysis of Cardiac Magnetic Resonance (CMR) and CT scans can identify early patterns of myocardial stiffening and coronary calcification, providing a crucial roadmap for predicting long-term Radiation-Induced Heart Disease (RIHD) [15,29,36].

According to the 2022 ESC Guidelines, the future of cardio-oncology lies in the development of digital health platforms where AI can integrate data from wearable devices, serial biomarkers, and advanced imaging to provide a continuous, real-time “cardiovascular risk trajectory” for each patient [4,14]. As emphasized by the JACC Council, these technological advancements will be essential to manage the complex needs of the global population of cancer survivors, ensuring that life-saving oncological treatments are not overshadowed by preventable cardiovascular morbidity [3,35,52].

## 11. Discussion

The rapid evolution of oncological therapies has fundamentally shifted the clinical focus from acute survival to long-term survivorship management. As underscored by the 2024 Cancer Statistics, the growing population of cancer survivors—exceeding 18 million in the US alone—highlights an urgent need for standardized cardio-oncological care to mitigate the “competing risks” of cancer recurrence and cardiovascular morbidity [3,4]. The evidence synthesized in this review demonstrates that chemotherapy-induced cardiotoxicity is not a single clinical entity but a heterogeneous spectrum of molecular insults, ranging from the permanent DNA-Top2b-mediated damage of anthracyclines to the transient, often reversible, signaling disruptions caused by HER2 inhibitors [3,7,22].

A critical point of discussion is the diagnostic paradigm shift from ejection fraction monitoring to myocardial deformation imaging. While traditional LVEF assessment remains the standard for defining heart failure with reduced ejection fraction (HFrEF), it often fails to detect subclinical injury during the “therapeutic window” where intervention is most effective [12,23,40]. The SUCCOUR trial provided definitive evidence in this regard, demonstrating that a management strategy guided by Global Longitudinal Strain (GLS)—initiating cardioprotection when a >15% relative reduction is detected—is superior to LVEF-based monitoring in preventing a subsequent clinical decline in cardiac function [18,32,40]. This proactive approach is further supported by the PRADA and CECCY trials, which validate the use of candesartan and carvedilol as effective prophylactic agents to preserve myocardial integrity during high-risk regimens [46,48].

The pathophysiology of cardiotoxicity in specialized populations, such as pediatric survivors and the elderly, further complicates the clinical landscape. In pediatric cases, the mismatch between somatic growth and myocardial mass gain—the “thin-walled” cardiomyopathy model—creates a lifelong vulnerability to hemodynamic stress [26,30]. Contrastingly, in geriatric patients, the primary challenge is the “unmasking” of pre-existing diastolic dysfunction (HFpEF) by newer agents like Tyrosine Kinase Inhibitors (TKIs) and Immune Checkpoint Inhibitors (ICIs) [11,41]. The management of these patients requires a sophisticated understanding of the “thrombo-hemorrhagic paradox” seen with Ibrutinib, where the risk of atrial fibrillation must be balanced against the drug’s inhibitory effect on platelet aggregation [27,33,42,43,52,53].

Moreover, the emergence of immunotherapy has introduced a high-acuity phenotype of cardiotoxicity, exemplified by ICI-induced myocarditis and CAR-T related cytokine release syndrome (CRS) [25,54]. Unlike the cumulative dose-dependent toxicity of anthracyclines, these events are often idiosyncratic and characterized by a rapid, lethal progression if not identified early through daily monitoring of biomarkers like Troponin and NT-proBNP [14,24,37]. The mandatory use of high-dose corticosteroids, such as Methylprednisolone, represents a cornerstone of management, yet it underscores the necessity for multidisciplinary cardio-oncology teams to balance immunosuppression with oncological efficacy [4,23,24,55].

Finally, the future of the field lies in the integration of artificial intelligence (AI) and radiomics. By synthesizing polygenic risk scores (e.g., the RARG variant) with high-throughput imaging features, AI can provide a personalized “cardiovascular risk trajectory” that was previously unattainable [19,32,40]. This aligns with the 2022 ESC Guidelines, which advocate for a stratified surveillance model based on individual baseline risk scores like HFA-ICOS [3,4]. In conclusion, the transition toward precision cardio-oncology, supported by robust clinical trial evidence and emerging technologies, is essential to fulfill the promise of modern oncology while ensuring the long-term cardiovascular health of cancer survivors [4,6,20,52,56].

## 12. Limitations

Despite the structured approach, this review is subject to several limitations. First, the field of cardio-oncology is evolving rapidly; the recent approval of next-generation targeted agents (2024–2025) means that long-term cardiovascular safety data for some molecules are still emerging. Second, significant heterogeneity exists among clinical trials regarding the definitions of cardiotoxicity and the thresholds used for LVEF decline, which complicates the direct comparison of different cardioprotective interventions. Third, the literature search was restricted to English-language publications, which may introduce a geographic selection bias. Finally, while we emphasize the role of Artificial Intelligence and radiomics, these technologies are still largely in the validation phase and have not yet reached universal clinical implementation.

## 13. Conclusions

The complex and evolving interplay between innovative cancer therapies and cardiac function necessitates a comprehensive, multidisciplinary approach that integrates the expertise of oncologists, cardiologists, and researchers. A deep understanding of the diverse pathophysiological mechanisms—ranging from direct DNA damage to immune-mediated inflammation—and the resulting clinical phenotypes enables the development of targeted prevention and personalized management strategies. This integrated model of care is essential for improving both oncological outcomes and long-term cardiovascular health. Future research efforts should prioritize the refinement of predictive tools, the optimization of novel cardioprotective therapies, and the expansion of international cardio-oncology collaborations. Only through such a concerted effort can the medical community ensure that the hard-won victory of cancer survival does not come at the devastating cost of long-term, irreversible cardiac health impairment.

## Figures and Tables

**Table 1:** Summary of anticancer agents, mechanisms, and clinical management.

Therapeutic Class	Key Agents	Molecular Mechanism	HF Phenotype	Management and Prevention	References
**Anthracyclines**	Doxorubicin, Epirubicin	Topoisomerase IIβ inhibition; ROS production; DNA double-strand breaks	HFrEF (Irreversible/Type I)	Dexrazoxane; ACE-i/ARBs; Beta-blockers; SGLT2i	[7,12,17,18,19,20]
**HER2 Inhibitors**	Trastuzumab, Pertuzumab	Blockade of ErbB2/NRG-1 survival signaling	HFrEF (Often reversible/Type II)	LVEF monitoring; GDMT; “Treat-through” strategies	[14,21,22]
**VEGF Inhibitors (TKIs)**	Sunitinib, Sorafenib	Endothelial dysfunction; NO reduction; Hypertension	HFpEF/Acute HFrEF	Strict BP control (<130/80); ACE-i; CCBs	[16,23]
**Immune Checkpoint Inhibitors (ICIs)**	Nivolumab, Pembrolizumab	T-cell hyperactivation; Molecular mimicry	Myocarditis/Arrhythmias	High-dose Steroids; Abatacept; Telemetry	[24,25]
**CAR-T Cell Therapy**	Axicabtagene ciloleucel	Cytokine Release Syndrome (IL-6, TNF-α surge)	Stress Cardiomyopathy/Hypotension	Tocilizumab; Corticosteroids; IL-6 monitoring	[26]
**BTK Inhibitors**	Ibrutinib	Off-target CSK inhibition in atria	Atrial Fibrillation	Anticoagulation (DOACs); Selectivity assessment	[27,28]
**Radiation Therapy**	Mediastinal RT	Microvascular injury; TGF-β mediated fibrosis	HFpEF/Valvular Disease	Long-term echo surveillance; Risk factor control	[10,15,29]

**Table 2:** Distinctive Features of HFrEF vs. HFpEF in Cardio-Oncology.

Feature	HFrEF (Reduced EF)	HFpEF (Preserved EF)	References
**LVEF Threshold**	<40%	≥50%	[4,10]
**Pathophysiology**	Cardiomyocyte death, myofibrillar loss, and chamber dilation	Ventricular stiffness, interstitial fibrosis, and impaired relaxation	[5,11]
**Primary Mechanism**	Direct cytotoxicity (e.g., Anthracyclines Type I injury)	Microvascular rarefaction and systemic inflammation (e.g., TKIs, RT)	[7,15,16]
**Echo Findings**	Reduced Global Longitudinal Strain (GLS) and low LVEF	Diastolic dysfunction (high E/e′ ratio) and Left Atrial enlargement	[14,32]
**Cardiac Geometry**	Eccentric remodeling (thin, dilated walls)	Concentric remodeling (thickened, stiff walls)	[5,6,22,33]
**Common Drug Triggers**	Anthracyclines, Trastuzumab, Proteasome inhibitors	TKIs (Sunitinib), VEGF inhibitors, Mediastinal Radiation	[2,7,15,16,21]
**Therapeutic Pillar**	GDMT (Beta-blockers, ACE-i/ARNI, MRAs, SGLT2i)	Control of comorbidities (HTN), SGLT2i, Diuretics for congestion	[12,17,34]

**Table 3:** Comparative Analysis of Pediatric vs. Geriatric Cardio-Oncology.

Feature	Pediatric Cardio-Oncology	Geriatric Cardio-Oncology	References
**Primary Mechanism**	Impaired myocardial growth; failure in cardiomyocyte proliferation.	Accelerated myocardial fibrosis and reduced beta-adrenergic sensitivity.	[11,16,26]
**Cardiac Phenotype**	Thin-walled cardiomyopathy; inadequate mass-to-BSA ratio.	Left ventricular hypertrophy and arterial stiffness.	[26,30,39]
**Typical HF Model**	Late-onset HFrEF (often decades after treatment).	Acute or subacute HFpEF (unmasked by therapy).	[26,35]
**Main Culprits**	High-dose Anthracyclines; Mediastinal Radiotherapy.	Tyrosine Kinase Inhibitors (TKIs); Immune Checkpoint Inhibitors (ICIs).	[9,15,19,25]
**Genomic Influence**	Coding variants in the RARG gene.	Polygenic risk scores for age-related cardiac decline.	[19,40]
**Clinical Challenge**	Lifelong competing risks; impaired cardiac reserve growth.	Polypharmacy and CYP3A4-mediated drug–drug interactions.	[26,39]
**Surveillance Focus**	Long-term serial Echo/CMR tracking mass-to-volume ratio.	Baseline risk stratification (HFA-ICOS) and comorbidity management.	[4,30,36,38]

**Table 4:** Cardiovascular and Hemostatic Challenges of Ibrutinib Therapy.

Clinical Domain	Effect of Ibrutinib	Pathophysiological Mechanism	Clinical Implication	References
**Electrophysiology**	Atrial Fibrillation (AF)	“Off-target” inhibition of CSK; altered calcium handling in cardiomyocytes.	Incidence up to 15%; requires rhythm/rate control strategies.	[27,42]
**Hemostasis**	Impaired Platelet Aggregation	Blockade of BTK and Tec kinases in collagen-induced signaling.	Increased risk of mucocutaneous bleeding and epistaxis.	[27,28]
**Combined Risk**	Thrombo-Hemorrhagic Paradox	Simultaneous embolic risk from AF and bleeding risk from platelet dysfunction.	Complex anticoagulation management; conflict between CHA_2_DS_2_-VASc and bleeding.	[27,28]
**Pharmacology**	Drug–Drug Interactions	Metabolism via the CYP3A4 hepatic pathway.	Potentiation of toxicity with calcium channel blockers or azoles.	[4,27]
**Therapeutic Evolution**	Next-Generation Selectivity	Development of Acalabrutinib and Zanubrutinib with higher BTK specificity.	Significantly lower incidence of cardiotoxicity and bleeding.	[27]

**Table 5:** Manifestations and Surveillance of Radiation-Induced Heart Disease (RIHD).

Complication	Pathophysiological Mechanism	Clinical Presentation	References
**Coronary Artery Disease**	Accelerated atherosclerosis and ostial stenosis; microvascular rarefaction.	Angina, Myocardial Infarction (often with late onset).	[15,23,29]
**Valvular Heart Disease**	Progressive calcification and fibrosis of the valves.	Valve stenosis or regurgitation; progressive heart failure.	[4,15,38]
**Pericardial Disease**	Acute inflammation or chronic constrictive pericarditis.	Pericardial effusion; chest pain; chronic constriction.	[23,36]
**Conduction System**	Fibrosis of the sinoatrial (SA) or atrioventricular (AV) nodes.	Heart blocks, sick sinus syndrome, or arrhythmias.	[4,15,42]
**Myocardial Fibrosis**	Direct tissue injury leading to diastolic dysfunction (HFpEF).	Reduced exercise tolerance; restrictive cardiomyopathy.	[15,23,36]

**Table 6:** Landmark Clinical Trials in Cardio-Oncology Prophylaxis and Monitoring.

Clinical Trial	Drug/Strategy Evaluated	Primary Findings and Cardiovascular Outcome	References
**PRADA**	Candesartan vs. Metoprolol	Candesartan attenuated LVEF decline; Metoprolol primarily reduced troponin spikes.	[44,46]
**SUCCOUR**	GLS-guided therapy	Strain-guided initiation of cardioprotection prevents late-onset LVEF reduction.	[18,47]
**CECCY**	Carvedilol	Reduced subclinical myocardial injury and improved diastolic function during Doxorubicin.	[48,49]
**ICOS-ONE**	Enalapril (Early vs. Late)	Supported early ACE-inhibitor initiation to prevent anthracycline-induced cardiotoxicity.	[12,13]
**HERA (8-year)**	Trastuzumab monitoring	Established the long-term safety profile and incidence of cardiac events in HER2+ survivors.	[21]
**CECCY (Follow-up)**	Lisinopril/Carvedilol	Comparative efficacy in preventing Trastuzumab-induced cardiotoxicity in breast cancer.	[48]

**Table 7:** Clinical Protocols for Cardiovascular Monitoring and Management of Suspected Toxicity.

Monitoring Phase	Clinical Action and Diagnostic Criteria	Rationale and Guidance	References
**Baseline Risk**	HFA-ICOS score; ECG; 2D-Echo for LVEF; biomarkers (Troponin, NT-proBNP).	Establishes surveillance frequency based on pre-existing cardiac risk.	[4,30,39]
**Serial Surveillance**	Periodic Echo and biomarkers during treatment, frequency based on drug type and dose.	Enables detection of subclinical myocardial injury during peak toxicity exposure.	[4,14,30]
**Suspected Toxicity**	Triggered by LVEF drop >10% to <50%, GLS reduction > 15%, or significant biomarker rise.	Defines thresholds for urgent diagnostic escalation and multidisciplinary review.	[4,18,23]
**Diagnostic Confirmation**	CMR (T2-mapping for edema, LGE for fibrosis); Stress Echo for ischemia.	Gold standard for tissue characterization and differential diagnosis (e.g., ICI-myocarditis vs. stress CMP).	[29,38]
**Intervention Strategy**	ACE-inhibitors (Enalapril), Beta-blockers (Carvedilol); Corticosteroids for ICI-related myocarditis.	Pharmacologic support to promote ventricular recovery and maintain oncologic treatment continuity.	[12,13]
**Follow-up**	Long-term monitoring (5–10 years post-therapy) for late-onset cardiotoxicity.	Critical for survivors exposed to anthracyclines, radiotherapy, or targeted agents.	[26,30]

**Table 8:** AI Domains in Cardiovascular Care.

AI Domain	Clinical Application	Impact on Patient Care	References
**Deep Learning (Computer Vision)**	Automated echocardiographic segmentation and LVEF calculation.	Reduces inter-observer variability and enhances reproducibility of functional monitoring.	[8,32]
**Automated Strain Analysis**	Real-time Global Longitudinal Strain (GLS) tracking via speckle-tracking.	Facilitates early detection of subclinical CTRCD as advocated by the SUCCOUR trial.	[18,32]
**Radiomics**	Quantitative feature extraction from CT and CMR scans.	Identifies subtle tissue changes and predicts long-term Radiation-Induced Heart Disease.	[15,29,36]
**Predictive Analytics (ML)**	Integration of “omics” data (e.g., RARG variant) with clinical parameters.	Enables personalized polygenic risk scoring and individualized surveillance protocols.	[19,40]
**Digital Health Integration**	Synthesis of data from wearables, biomarkers, and serial imaging.	Provides a continuous, real-time cardiovascular risk trajectory throughout survivorship.	[4,14,35]

## Data Availability

No new data were created or analyzed in this study. Data sharing is not applicable to this article.

## Abbreviations

The following abbreviations are used in this manuscript:

ACE-i	Angiotensin-Converting Enzyme inhibitor
AF	Atrial Fibrillation
AI	Artificial Intelligence
AIC	Anthracycline-Induced Cardiotoxicity
AMPK	AMP-activated Protein Kinase
BSA	Body Surface Area
BTK	Bruton Tyrosine Kinase
CMR	Cardiac Magnetic Resonance
CSK	C-terminal Src Kinase
CTRCD	Cancer Therapy-Related Cardiac Dysfunction
DIBH	Deep Inspiration Breath Hold
DOACs	Direct Oral Anticoagulants
eNOS	endothelial Nitric Oxide Synthase
GLS	Global Longitudinal Strain
HFpEF	Heart Failure with preserved Ejection Fraction
HFrEF	Heart Failure with reduced Ejection Fraction
ICIs	Immune Checkpoint Inhibitors
IMRT	Intensity-Modulated Radiation Therapy
LGE	Late Gadolinium Enhancement
LVEF	Left Ventricular Ejection Fraction
MHD	Mean Heart Dose
NO	Nitric Oxide
NOACs	Non-vitamin K antagonist Oral Anticoagulants
NRG1	Neuregulin-1
NT-proBNP	N-terminal pro-B-type Natriuretic Peptide
RARG	Retinoic Acid Receptor Gamma
RIHD	Radiation-Induced Heart Disease
ROS	Reactive Oxygen Species
RT	Radiation Therapy
TKIs	Tyrosine Kinase Inhibitors
Top2b	Topoisomerase II-beta
VEGF	Vascular Endothelial Growth Factor

## References

[1] Lefrak E.A., Pitha J., Rosenheim S., Gottlieb J.A. (1973). A clinicopathologic analysis of adriamycin cardiotoxicity. Cancer.

[2] Ewer M.S., Ewer S.M. (2015). Cardiotoxicity of anticancer treatments. Nat. Rev. Cardiol..

[3] Siegel R.L., Giaquinto A.N., Jemal A. (2024). Cancer statistics, 2024. CA Cancer J. Clin..

[4] Lyon A.R., López-Fernández T., Couch L.S., Asteggiano R., Aznar M.C., Bergler-Klein J., Boriani G., Cardinale D., Cordoba R., Cosyns B. (2022). 2022 ESC Guidelines on cardio-oncology developed in collaboration with the European Hematology Association (EHA), the European Society for Therapeutic Radiology and Oncology (ESTRO) and the International Cardio-Oncology Society (IC-OS). Eur. Heart J..

[5] Herrmann J. (2024). Clinical Cardio-Oncology.

[6] Herrmann J. (2019). From trends to transformation: Where cardio-oncology is to make a difference. Eur. Heart J..

[7] Zhang S., Liu X., Bawa-Khalfe T., Lu L.S., Lyu Y.L., Liu L.F., Yeh E.T. (2012). Identification of the molecular basis of doxorubicin-induced cardiotoxicity. Nat. Med..

[8] Schimmel K.J., Richel D.J., van den Brink R.B., Guchelaar H.J. (2004). Cardiotoxicity of cytotoxic drugs. Cancer Treat. Rev..

[9] Moslehi J. (2013). The cardiovascular perils of cancer survivorship. N. Engl. J. Med..

[10] Zamorano J.L., Lancellotti P., Rodriguez Muñoz D., Aboyans V., Asteggiano R., Galderisi M., Habib G., Lenihan D.J., Lip G.Y.H., Lyon A.R. (2016). 2016 ESC Position Paper on cancer treatments and cardiovascular toxicity developed under the auspices of the ESC Committee for Practice Guidelines: The Task Force for cancer treatments and cardiovascular toxicity of the European Society of Cardiology (ESC). Eur. Heart J..

[11] Kourek C., Touloupaki M., Rempakos A., Loritis K., Tsougkos E., Paraskevaidis I., Briasoulis A. (2022). Cardioprotective Strategies from Cardiotoxicity in Cancer Patients: A Comprehensive Review. J. Cardiovasc. Dev. Dis..

[12] Cardinale D., Colombo A., Bacchiani G., Tedeschi I., Meroni C.A., Veglia F., Civelli M., Lamantia G., Colombo N., Curigliano G. (2015). Early detection of anthracycline cardiotoxicity and improvement with heart failure therapy. Circulation.

[13] Cardinale D., Ciceri F., Latini R., Franzosi M.G., Sandri M.T., Civelli M., Cucchi G., Menatti E., Mangiavacchi M., Cavina R. (2018). Anthracycline-induced cardiotoxicity: A multicenter randomised trial comparing two strategies for guiding prevention with enalapril: The International CardioOncology Society-one trial. Eur. J. Cancer..

[14] Ky B., Putt M., Sawaya H., French B., Januzzi J.L., Sebag I.A., Plana J.C., Cohen V., Banchs J., Carver J.R., Wiegers S.E. (2014). Early increases in multiple biomarkers predict subsequent cardiotoxicity in patients with breast cancer treated with doxorubicin, taxanes, and trastuzumab. J. Am. Coll. Cardiol..

[15] Darby S.C., Ewertz M., McGale P., Bennet A.M., Blom-Goldman U., Brønnum D., Correa C., Cutter D., Gagliardi G., Gigante B. (2013). Risk of ischemic heart disease in women after radiotherapy for breast cancer. N. Engl. J. Med..

[16] de Wit S., Glen C., de Boer R.A., Lang N.N. (2023). Mechanisms shared between cancer, heart failure, and targeted anti-cancer therapies. Cardiovasc. Res..

[17] Sabatino J., De Rosa S., Tammè L., Iaconetti C., Sorrentino S., Polimeni A., Mignogna C., Amorosi A., Spaccarotella C., Yasuda M. (2020). Empagliflozin prevents doxorubicin-induced myocardial dysfunction. Cardiovasc. Diabetol..

[18] Thavendiranathan P., Negishi T., Somerset E., Negishi K., Penicka M., Lemieux J., Aakhus S., Miyazaki S., Shirazi M., Galderisi M. (2021). Strain-Guided Management of Potentially Cardiotoxic Cancer Therapy. J. Am. Coll. Cardiol..

[19] Skitch A., Mital S., Mertens L., Liu P., Kantor P., Grosse-Wortmann L., Manlhiot C., Greenberg M., Nathan P.C. (2017). Novel approaches to the prediction, diagnosis and treatment of cardiac late effects in survivors of childhood cancer: A multi-centre observational study. BMC Cancer.

[20] Vacharanukrauh P., Miller K.J., Alif S.M., Grace F., Rahman M.A. (2025). Pharmacological interventions for anthracycline-induced cardiotoxicity in breast cancer: A systematic review and meta-analysis of randomized controlled trials. Breast Cancer Res. Treat..

[21] Cameron D., Piccart-Gebhart M.J., Gelber R.D., Procter M., Goldhirsch A., de Azambuja E., Castro GJr Untch M., Smith I., Gianni L., Baselga J. (2017). 11 years’ follow-up of trastuzumab after adjuvant chemotherapy in HER2-positive early breast cancer: Final analysis of the HERceptin Adjuvant (HERA) trial. Lancet.

[22] Pivot X., Romieu G., Debled M., Pierga J.Y., Kerbrat P., Bachelot T., Lortholary A., Espié M., Fumoleau P., Serin D. (2019). 6 months versus 12 months of adjuvant trastuzumab in early breast cancer (PHARE): Final analysis of a multicentre, open-label, phase 3 randomised trial. Lancet.

[23] Ganatra S., Barac A., Armenian S., Cambareri C., Denlinger C.S., Dent S.F., Hayek S., Ky B., Leja M., Lucas C.H. (2026). Diagnosis and Management of Cardiovascular Adverse Effects of Targeted Oncology Therapies: Bruton’s Tyrosine Kinase, Immune Checkpoint, and Vascular Endothelial Growth Factor Inhibitors: 2025 ACC Concise Clinical Guidance: A Report of the American College of Cardiology Solution Set Oversight Committee. J. Am. Coll. Cardiol..

[24] Bonaca M.P., Olenchock B.A., Salem J.E., Wiviott S.D., Ederhy S., Cohen A., Stewart G.C., Choueiri T.K., Di Carli M., Allenbach Y. (2019). Myocarditis in the Setting of Cancer Therapeutics: Proposed Case Definitions for Emerging Clinical Syndromes in Cardio-Oncology. Circulation.

[25] Mahmood S.S., Fradley M.G., Cohen J.V., Nohria A., Reynolds K.L., Heinzerling L.M., Sullivan R.J., Damrongwatanasuk R., Chen C.L., Gupta D. (2018). Myocarditis in Patients Treated With Immune Checkpoint Inhibitors. J. Am. Coll. Cardiol..

[26] Mulrooney D.A., Hyun G., Ness K.K., Ehrhardt M.J., Yasui Y., Duprez D., Howell R.M., Leisenring W.M., Constine L.S., Tonorezos E. (2020). Major cardiac events for adult survivors of childhood cancer diagnosed between 1970 and 1999: Report from the Childhood Cancer Survivor Study cohort. BMJ.

[27] Spadafora L., Russo F., Bukowska-Olech E., Panichella G., Garofalo M., Cacciatore S., Sabouret P., Sarto G., Simeone B., Rocco E. (2026). Cardiovascular Safety of Bruton Tyrosine Kinase Inhibitors: From Ibrutinib to Next-Generation Agents. Am. J. Cardiovasc. Drugs.

[28] Delluc A., Wang T.F., Yap E.S., Ay C., Schaefer J., Carrier M., Noble S. (2019). Anticoagulation of cancer patients with non-valvular atrial fibrillation receiving chemotherapy: Guidance from the SSC of the ISTH. J. Thromb. Haemost..

[29] Bergom C., Bradley J.A., Ng A.K., Samson P., Robinson C., Lopez-Mattei J., Mitchell J.D. (2021). Past, Present, and Future of Radiation-Induced Cardiotoxicity: Refinements in Targeting, Surveillance, and Risk Stratification. JACC Cardio Oncol..

[30] Armenian S.H., Lacchetti C., Barac A., Carver J., Constine L.S., Denduluri N., Dent S., Douglas P.S., Durand J.B., Ewer M. (2017). Prevention and Monitoring of Cardiac Dysfunction in Survivors of Adult Cancers: American Society of Clinical Oncology Clinical Practice Guideline. J. Clin. Oncol..

[31] Vejpongsa P., Yeh E.T. (2014). Prevention of anthracycline-induced cardiotoxicity: Challenges and opportunities. J. Am. Coll. Cardiol..

[32] López-Fernández T., Martín-García A., Roldán Rabadán I., Mitroi C., Mazón Ramos P., Díez-Villanueva P., Escobar Cervantes C., Alonso Martín C., Alonso Salinas G.L., Arenas M. (2019). Atrial Fibrillation in Active Cancer Patients: Expert Position Paper and Recommendations. Rev. Española Cardiol. (Engl. Ed.).

[33] Tana M., Piccinini R., Moffa L., Tana C. (2024). Heart Failure with Preserved Ejection Fraction and Cardiac Amyloidosis in the Aging Heart. Int. J. Mol. Sci..

[34] Bhalraam U., Veerni R.B., Paddock S., Meng J., Piepoli M., López-Fernández T., Tsampasian V., Vassiliou V.S. (2026). Impact of sodium-glucose cotransporter-2 inhibitors on heart failure outcomes in cancer patients and survivors: A systematic review and meta-analysis. Eur. J. Prev. Cardiol..

[35] Curigliano G., Lenihan D., Fradley M., Ganatra S., Barac A., Blaes A., Herrmann J., Porter C., Lyon A.R., Lancellotti P. (2020). Management of cardiac disease in cancer patients throughout oncological treatment: ESMO consensus recommendations. Ann. Oncol..

[36] Madan N., Lucas J., Akhter N., Collier P., Cheng F., Guha A., Zhang L., Sharma A., Hamid A., Ndiokho I. (2022). Artificial intelligence and imaging: Opportunities in cardio-oncology. Am. Heart J. Plus..

[37] Totzeck M., Michel L., Lin Y., Herrmann J., Rassaf T. (2022). Cardiotoxicity from chimeric antigen receptor-T cell therapy for advanced malignancies. Eur. Heart J..

[38] Plana J.C., Galderisi M., Barac A., Ewer M.S., Ky B., Scherrer-Crosbie M., Ganame J., Sebag I.A., Agler D.A., Badano L.P. (2014). Expert consensus for multimodality imaging evaluation of adult patients during and after cancer therapy: A report from the American Society of Echocardiography and the European Association of Cardiovascular Imaging. J. Am. Soc. Echocardiogr..

[39] Al-Kindi S.G., Oliveira G.H. (2016). Prevalence of Preexisting Cardiovascular Disease in Patients with Different Types of Cancer: The Unmet Need for Onco-Cardiology. Mayo Clin. Proc..

[40] Aminkeng F., Bhavsar A.P., Visscher H., Rassekh S.R., Li Y., Lee J.W., Brunham L.R., Caron H.N., van Dalen E.C., Kremer L.C. (2015). A coding variant in RARG confers susceptibility to anthracycline-induced cardiotoxicity in childhood cancer. Nat. Genet..

[41] Doukas P.G., Patel R.N., Venkatesh V., Khan S.S., Baldridge A., Akhter N. (2021). Cardiac risk stratification of breast cancer patients in a cardio-oncology clinic. Breast Cancer Res. Treat..

[42] Mery B., Guichard J.B., Guy J.B., Vallard A., Barthelemy J.C., Da Costa A., Magné N., Bertoletti L. (2017). Atrial fibrillation in cancer patients: Hindsight, insight and foresight. Int. J. Cardiol..

[43] Tana M., Tana C., Rossi D., Mantini C., Gallina S., Ricci F., Porreca E. (2024). Thromboembolic and bleeding risk in cardiac amyloidosis. J. Thromb. Haemost..

[44] Gulati G., Heck S.L., Ree A.H., Hoffmann P., Schulz-Menger J., Fagerland M.W., Gravdehaug B., von Knobelsdorff-Brenkenhoff F., Bratland Å., Storås T.H. (2016). Prevention of cardiac dysfunction during adjuvant breast cancer therapy (PRADA): A 2 × 2 factorial, randomized, placebo-controlled, double-blind clinical trial of candesartan and metoprolol. Eur. Heart J..

[45] Gulati G. (2022). Cardioprotection in breast cancer patients: One size fits all?. Eur. Heart J..

[46] Heck S.L., Mecinaj A., Ree A.H., Hoffmann P., Schulz-Menger J., Fagerland M.W., Gravdehaug B., Røsjø H., Steine K., Geisler J. (2021). Prevention of Cardiac Dysfunction During Adjuvant Breast Cancer Therapy (PRADA): Extended Follow-Up of a 2 × 2 Factorial, Randomized, Placebo-Controlled, Double-Blind Clinical Trial of Candesartan and Metoprolol. Circulation.

[47] Oikonomou E.K., Kokkinidis D.G., Kampaktsis P.N., Amir E.A., Marwick T.H., Gupta D., Thavendiranathan P. (2019). Assessment of Prognostic Value of Left Ventricular Global Longitudinal Strain for Early Prediction of Chemotherapy-Induced Cardiotoxicity: A Systematic Review and Meta-analysis. JAMA Cardiol..

[48] Avila M.S., Ayub-Ferreira S.M., de Barros Wanderley M.R., Jr das Dores Cruz F., Gonçalves Brandão S.M., Rigaud V.O.C., Higuchi-Dos-Santos M.H., Hajjar L.A., Kalil Filho R., Hoff P.M. (2018). Carvedilol for Prevention of Chemotherapy-Related Cardiotoxicity: The CECCY Trial. J. Am. Coll. Cardiol..

[49] Bosch X., Rovira M., Sitges M., Domènech A., Ortiz-Pérez J.T., de Caralt T.M., Morales-Ruiz M., Perea R.J., Monzó M., Esteve J. (2013). Enalapril and carvedilol for preventing chemotherapy-induced left ventricular systolic dysfunction in patients with malignant hemopathies: The OVERCOME trial (preventiOn of left Ventricular dysfunction with Enalapril and caRvedilol in patients submitted to intensive ChemOtherapy for the treatment of Malignant hEmopathies). J. Am. Coll. Cardiol..

[50] Ravera F., Gilardi N., Ballestrero A., Zoppoli G. (2025). Applications, challenges and future directions of artificial intelligence in cardio-oncology. Eur. J. Clin. Investig..

[51] Ștefan M.F., Magda L.Ș., Vinereanu D. (2026). Artificial Intelligence and the Expanding Universe of Cardio-Oncology: Beyond Detection Toward Prediction and Prevention of Therapy-Related Cardiotoxicity-A Comprehensive Review. Diagnostics.

[52] Tana M., Panarese A., Tana C., Mantini C., Caulo M., Ricci F., Porreca E. (2023). Clinical and Cardiovascular Magnetic Resonance Imaging Features of Cardiac Amyloidosis. Rev. Cardiovasc. Med..

[53] Tana M., Tana C., Palmiero G., Mantini C., Caulo M., Gallina S., Ricci F., Porreca E. (2023). Imaging findings of right cardiac amyloidosis: Impact on prognosis and clinical course. J. Ultrasound.

[54] Lenneman C.G., Sawyer D.B. (2016). Cardio-Oncology: An Update on Cardiotoxicity of Cancer-Related Treatment. Circ. Res..

[55] Elbl L., Hrstkova H., Tomaskova I., Michalek J. (2006). Late anthracycline cardiotoxicity protection by dexrazoxane (ICRF-187) in pediatric patients: Echocardiographic follow-up. Support Care Cancer.

[56] Gilchrist S.C., Barac A., Ades P.A., Alfano C.M., Franklin B.A., Jones L.W., La Gerche A., Ligibel J.A., Lopez G., Madan K. (2019). Cardio-Oncology Rehabilitation to Manage Cardiovascular Outcomes in Cancer Patients and Survivors: A Scientific Statement From the American Heart Association. Circulation.

